# Magnitude of admission, premature mortality and potential years of life lost due to acute diseases among pediatric patients admitted to public hospitals in Jimma City, Southwest Ethiopia

**DOI:** 10.11604/pamj.2022.43.135.31541

**Published:** 2022-11-14

**Authors:** Sheka Shemsi Seid, Shemsedin Amme Ibro, Abdulwahid Awol Ahmed, Gaius Rexford Kunzong

**Affiliations:** 1 School of Nursing, Faculty of Health Sciences, Institute of Health, Jimma University, Jimma, Ethiopia,; 2 Department of Obstetrics and Gynecology, Tamale Teaching Hospital, Tamale, Ghana

**Keywords:** Premature mortality, admission, acute diseases, children

## Abstract

**Introduction:**

although evidence suggests recent reductions in infant and child mortality, little is known about the magnitude, and causes of pediatrics admission, premature mortality, and associated years of potential life lost among hospitalized children in Ethiopia, particularly in Jimma City.

**Methods:**

a retrospective cross-sectional study was conducted on hospital's care registries of pediatric patients who presented with acute disease over three years period, from September 7^th^, 2014, to September 10^th^, 2017, at Jimma Medical Canter and Shenen Gibe Hospital in Jimma City. The data were cleaned and imported to statistical package for the social sciences (SPSS) V.23.0 for descriptive statistical analysis.

**Results:**

a total of 7612 children were admitted to two public hospitals in Jimma City during the study period. Among them, 4457(58.6%) were males. The mean (SD) age of the children at admission was 4.1± (4.25) years. The major cause of admission was pneumonia in 2274 (29.9 %) children. The major causes of premature mortality were Pneumonia 36 (22.1%), sepsis 25 (15.3%), and severe acute malnutrition 25 (15.3%). A total of 9633 years were lost due to premature deaths, of which the majority 7663 (79.6%) were attributed to communicable and nutritional diseases. Pneumonia was responsible for the highest proportion of years of life lost 2178 (22.1%).

**Conclusion:**

it is indicated that the leading causes of hospital admissions and deaths were communicable and nutritional diseases. A significant number of years of life have been lost because of preventable and curable diseases. Therefore, early detection and initiation of an appropriate intervention could reduce the hospital´s burden and years of potential life lost due to these diseases.

## Introduction

Over the last 20 years, extensive progress has been made in reducing mortality among children aged less than 14 years. Despite these achievements and advancements in healthcare over the past two decades, the number of premature childhood deaths remains high. For instance, in 2017 alone, an estimated 6.3 million children and young adolescents died, mostly from preventable causes. The majority of these deaths occurred among neonates (2.5 million), while the remaining were infants, 1.6 million, children aged 12-59 months, 1.3 million, and children aged 5-14 years, 0.9 million [1]. According to the 2019 Ethiopian demographic health survey (EDHS), the neonatal, infant, and under-five mortality rates during the 5 years before the survey were 30, 43, and 55 deaths per 1,000 live births, respectively [2]. In other words, in Ethiopia, 1 in every 35 children dies within the first month, 1 in every 21 children dies before celebrating their first birthday, and 1 in every 15 children dies before reaching their fifth birthday [2]. Studies in several developing countries have shown that infectious diseases are the most common cause of admissions and mortality in hospital pediatrics wards; specifically, acute gastroenteritis, malaria, pneumonia, sepsis, meningitis, and malnutrition are the most common conditions [3-8]. Death that occurs before reaching the existing life expectancy of a particular country is called premature death, and it is the best single alternative measure to reflect differences in the health status of the population [9]. According to the World Health Organization (WHO) and Global Burden of Disease study (GBD), premature mortality is measured using the standard expected years of life lost (PYLL) [10].

Years of life lost due to premature death are also defined as dying before the ideal life span and are a good indicator of the disease's fatal burden [11]. In Ethiopia, the leading causes of premature death in the pediatric age group are lower respiratory infections, diarrheal diseases, and malaria [12]. The study of the causes of hospital admissions and the magnitude of premature deaths is a vital parameter for evaluating the quality of child healthcare and will be a significant input for modifying health policies. Furthermore, it helps to provide better quality care, hospital resource allocation, and institute adequate preventive measures [4,5]. Although knowing the causes of childhood admission and hospital outcomes enables the setting of appropriate priority and intervention planning [7,13], there is limited information regarding the magnitude and causes of pediatric admission, premature mortality, and associated years of potential life lost among hospitalized children in Ethiopia, particularly in Jimma City. Researchers believe that the paucity of information regarding the magnitude of acute presentation, and premature mortality critically hinder the design and rendering of appropriate care and preventive measures for acutely ill pediatric patients. Therefore, the present study aimed to assess the magnitude and causes of pediatric admissions, premature mortality, and associated years of potential life lost among pediatric patients admitted to public hospitals in Jimma City, Southwest Ethiopia.

## Methods

**Study design:** a facility-based retrospective cross-sectional study was conducted.

**Setting:** the study was conducted at Jimma Medical Center (JMC) and Shenen Gibe Hospital (SGH), located in Jimma City. Jimma City is located 352 km away towards the southwest of Addis Ababa. At the time of this study, the SGH and JMC were the only public hospitals in Jimma. Both hospitals provide pediatric outpatient and inpatient services. Jimma medical center is the only referral teaching hospital in the southwestern part of the country, serving a catchment area of 20 million people, whereas SGH is a regional hospital serving the population of Jimma and adjacent districts. The JMC has approximately 800 inpatient beds and the SGH has approximately 83 beds. Neither of these hospitals have a formal pediatric emergency department, however, pediatric patients with acute conditions are often evaluated at outpatient department services and admitted to the inpatient unit. There were four primary public health centers in Jimma, and these were not included in the present study because pediatric patients who attended the public health center with acute illness were likely transferred to either JMC or SGH.

**Population:** the study population included all pediatric patients admitted to Shenen Gibe Hospital and Jimma medical center with acute illness between September 7^th^, 2014, and September 11^th^, 2017. In the Ethiopian context, the population aged 0-14 years is considered a pediatric population.

**Inclusion and exclusion criteria:** the records of all pediatric patients who presented during the study period were reviewed. A patient presentation was classified as emergent if: i) the condition, if not diagnosed and treated, could lead to serious physical or mental disability or death within hours to days (e.g. shock, diarrhea, severe asthmatic attack, etc.); or ii) the condition could lead to acute decompensation, resulting in serious physical or mental disability or death within days. All patients who did not have a documented diagnosis or were recorded as “dead on arrival,” or “left before they were called/seen” were excluded. Patients who presented with non-acute conditions (e.g, osteoarthritis, dementia) were also excluded.

**Data source and data collection methods:** paper-based records of admission of children aged greater than one month and less than fourteen years (≥1 month and ≤14 years) admitted to pediatric inpatient units of JMC and SGH were used as source of data. Data were extracted from each case registry and entered into a prepared Microsoft Excel spreadsheet by the department's head of service and one assistant working in the department. The information obtained included age, sex, residential address, date of service, diagnosis, and clinical outcomes.

**Data analysis:** the 2017 GBD disease catalog was used to manually classify the causes of admission on a Microsoft Excel spreadsheet into four categories [3]. The first category contains three broad aggregates: communicable diseases and nutritional disorders (CNDs), non-communicable diseases (NCD), and injuries, which were used to understand the relative contribution of each group of diseases to admission and mortality. These three broad aggregate categories were then sub-classified into another aggregate category (aggregated category II). For example: communicable diseases and nutritional disorders were further classified as infectious diseases and parasitic diseases, respiratory infections, and nutritional diseases, whereas NCDs were classified as cardiovascular, digestive diseases and etc. and injuries as intentional, unintentional, and unspecific injuries. Category II was further subclassified into disaggregated disease category (III). Infectious and parasitic diseases are sub-classified as acute febrile illness, diarrhea, etc. cardiovascular disease is sub-classified as congestive heart failure, rheumatic heart disease, etc. and unintentional injuries such as bite injury and blunt injury. Category III was then further classified as groups of specific diagnoses of illness or injury, such as limb fracture, malaria, measles, and pneumonia. The WHO list of disease catalog under each level of classification was modified based on their magnitude to identify the conditions of the disease that resulted in admissions and deaths.

**Statistical analysis:** the data classified on Microsoft Excel spreadsheets were cleaned and imported to SPSS Version 23.0 for further statistical analysis. Descriptive statistics were used to summarize demographic data, causes of admission and death, and potential years of life lost. The potential years of life lost (PPYLL) were calculated as follows.

**Potential years of life lost (PYLL):** is the year of potential life lost due to premature death; it quantifies the year that a person would have lived had he or she not died prematurely from a cause and is a measure of premature mortality that takes into account both the frequency of deaths and the age at which it occurs. Potential Years of life lost is calculated based on standardized life expectancy, obtained from the difference between life expectancy at birth and the actual year of death for this purpose, Ethiopian life expectancy at birth for the year 2017 was used. The formula used to calculate PYLL is: PYLL = N x L. Where: i) PYLL- is a potential year of life lost due to premature death; ii) N-is the number of deaths due to specific conditions; iii) L-is the standard life expectancy at the age at which death occurs. (Obtained from expectancy - an actual year of death) [3]. *In another way*: firstly, the years of life lost were obtained by subtracting the actual age at which death occurred from the standard life expectancy at birth (that is Ethiopian life expectancy at birth for the year 2017). Then, the obtained result was summed up to calculate the total years of life lost for the overall and or each group of illness diagnoses. For instance, to obtain PYLL for Lower respiratory infections, the difference between the standard life expectancy at birth (65.87) and the actual age at which the child was dead due to lower respiratory infections. A death occurring at five years of age due to respiratory disease is counted as 60.87 years of PYLL, which is obtained by subtracting 5 years from 65.87 years. To obtain PYLL from multiple dead cases, the calculated PYLL of each dead case will be summed up together.

**Premature mortality:** in this study, the premature mortality refers to a death that occurred before reaching the Ethiopian life expectancy at birth for the year 2017 which was 65.87 years.

**Ethical considerations:** before the commencement of the study, ethical clearance was obtained from Jimma University Institute of Health IRB on January 25^th^, 2018, with reference number IHRPGD/353/2018, granted an official letter of recommendation and ethical clearance after review of the study protocols and procedures, which were then accepted by the Jimma Municipal Health Bureau and Hospital Administrative Bodies. We guaranteed confidentiality by excluding names or any other personal identifiers from the data-collection sheets and reports. The identifier for each eligible subject was replaced by a code, and no master code exists that allows the research data to be linked with the identifiers.

## Results

A total of 7612 children were admitted to two public hospitals in Jimma City during the study period. The majority, 4457 (58.6%) were males. The mean (SD) age of the children was 4.1± (4.25) years, while children between 1-5 years accounted for 2932 (38.52%), followed by those under one-year 2165 (28.44%). More than half, 3991 (52.44%) were from Jimma City and 3757 (49.35%) were new admissions. Those admitted during 2014/15 accounted for 2758 (36.23%), while 3293 (43.26%), were admitted during the wet season. The majority, 7123 (93.60%) were admitted and diagnosed with a single illness. With regard to admission outcome, 7106 patients (93.30 %) had improved/discharged outcomes, whereas 163 patients (2.14%) died (Table 1).

**Table 1:** demographic characteristics of children admitted to the public hospitals in Jimma City, Southwest Ethiopia from September 11^th^, 2014, to September 10^th^, 2017 (2007 to 2009 E.C), N=7612

Variables		No	(%)
Age	<1years	2165	(28.44)
	1- ≤5 years	2932	(38.52)
	5- ≦ 9 years	1287	(16.90)
	10-14 years	1228	(16.13)
	Total	7612	(100)
Sex	Male	4457	(58.55)
	Female	3155	(41.44)
	Total	7612	(100)
Residency address	From Jimma	3992	(52.44)
	Out of Jimma	3620	(47.56 )
	Total	7612	(100)
Visit type	New	3757	(49.35)
	Repeat	138	(1.82)
	Missing	3717	(48.83)
	Total	7612	(100)
Season of visit	Wet seasons	3293	(43.26)
	Dry season	2477	(32.54)
	Rainy season	1842	(24.20)
	Total	7612	(100)
Year of visits	2014/2015 (2007 EFY)	2758	(36.23)
	2015/2016 (2008 EFY)	2701	(35.48)
	2016/2017 (2009 EFY)	2153	(28.28)
	Total	7612	(100)
Presence of comorbidities	No	7123	(93.60)
	Yes	489	(6.4)
	Total	7612	(100)
Patients outcomes on the disposition	Died	163	(2.14)
	Discharged (improved)	7106	(93.30)
	LAMA	130	1.70
	Others*	213	2.80
	**Total**	7612	(100)

**Aggregated causes of admission:** communicable and nutritional diseases (CNDs) accounted for the majority of admission, 5567 (73.1%); among these, 2709 (35.6%) were infectious and parasitic diseases. Non-communicable diseases (NCDs) accounted for 1051(13.8%) of the total admissions, of which two hundred fifty-four (3.3%) were attributed to cardiovascular diseases. Nine hundred and -four (13.1%) were admitted due to injuries, of which six hundred and twenty-five (8.2%) of them were unspecified, followed by unintentional injuries, which accounted for 354(4.7%) of the total admissions to pediatric wards (Table 2).

**Table 2:** causes of admission by aggregate disease category among patients admitted to public hospitals in Jimma City, Southwest Ethiopia from September 11^th^, 2014, to September 10^th^, 2017, N=7612

S/no	Aggregate disease category I	Aggregate category II	No	(%)
1	CNDs	Infectious and parasitic diseases	2709	(35.6)
		Respiratory Infectious	2323	(30.5)
		Nutritional deficiencies	535	(7.0)
		**TOTAL**	5567	(73.1)
2	NCDs	Cardiovascular diseases	254	(3.3)
		Digestive diseases	179	(2.4)
		Congenital anomalies	118	(1.6)
		Respiratory tract diseases	109	(1.4)
		Endocrine, blood, immune disorders	101	(1.3)
		Genitourinary diseases	96	(1.3)
		Neurological conditions	74	(1.0)
		Diseases of the skin and other medical conditions	45	(0.6)
		Diabetes mellitus and glycemic condition	36	(0.5)
		Malignant neoplasms	17	(0.2)
		Intentional injuries	15	(0.2)
		Mental and substance use disorders	11	(0.1)
		Musculoskeletal diseases	6	(0.1)
		Sense organ diseases	5	(0.1)
		**Total**	1051	(13.8)
3	Injuries	Unspecified injuries	625	(8.2)
		Unintentional injuries	354	(4.6)
		Intentional Injuries	15	(0.3)
		**Total**	994	(13.1)

CNDs: communicable and nutritional diseases; NCDs: non-communicable diseases

**Communicable diseases and nutritional diseases (CNDs) among children admitted to the pediatric wards of Jimma public hospitals:** of the 2709 (48.7%) cases of infectious and parasitic diseases, the most common reason for admission among the children was acute febrile illness 626 (11.2%), followed by diarrheal disease 545 (9.8%). Nutritional conditions accounted for 535 (9.6%) of the total admissions owing to communicable and nutritional diseases. The majority of the children were predominantly admitted due to lower respiratory infections 2276 (40.9%) as shown in Table 3.

**Table 3:** communicable and nutritional conditions among pediatric patients admitted to public hospitals in Jimma City, Southwest Ethiopia from September 11^th^, 2014, to September 10^th^, 2017, N=5567

Communicable and nutritional diseases	Category	No	(%)
Infectious and parasitic diseases	Acute febrile illness	626	(11.2)
	Diarrheal diseases	545	(9.8)
	Septicemia	385	(6.9)
	Childhood-cluster diseases	282	(5.1)
	Meningitis	236	(4.2)
	Other infectious diseases	209	(3.8)
	Abscess of internal	173	(3.1)
	Tuberculosis	84	(1.5)
	Parasitic and vector diseases	82	(1.5)
	Urinary tract infections	40	(0.7)
	HIV/AIDS	26	(0.5)
	Hepatitis	19	(0.3)
	Intestinal nematode infections	2	(0)
	**Total**	**2709**	**(48.7)**
Nutritional deficiencies	Nutritional deficiencies	**535**	**(9.6)**
Respiratory infectious	Lower respiratory infections	2276	(40.9)
	Upper respiratory infections	47	(0.8)
	Total	**2323**	**(41.7)**

**Non-communicable diseases (NCDs) among children admitted to pediatric wards in Jimma public hospital:** the major subcategory of non-communicable diseases was cardiovascular diseases, of which congestive heart failure accounted for 177 (16.8%) of the total admissions due to NCDs. Digestive system disease was the second predominant cause of admission in this subcategory; out of which acute abdomen accounted for 142 (13.5%) of the total admissions (Table 4).

**Table 4:** non-communicable diseases among patients admitted to public hospitals in Jimma City, Southwest Ethiopia from September 11^th^, 2014, to September 10^th^, 2017, N=1051

Non-communicable diseases	Category	No	(%)
Cardiovascular diseases	Congestive heart failure	177	(16.8)
	Rheumatic heart disease	51	(4.9)
	Other circulatory diseases	26	(2.5)
Congenital anomalies	Congenital heart anomalies	59	(5.60
	Anorectal malformation	14	(1.3)
	Other Misc. congenital anomalies	45	(4.3)
Digestive diseases	Acute abdomen	142	(13.5)
	Other miscellaneous digestive diseases	37	(3.5)
Endocrine, blood, and immune disorders	Anemia	60	(5.7)
	Diabetes mellitus	36	(3.4)
	Bleeding disorder and electrolyte deficits	41	(3.9)
Genitourinary diseases	Kidney diseases	89	(8.5)
	Other genitourinary diseases	7	(0.7)
Neurological conditions	Epilepsy	35	(3.3)
	Acute flaccid paralysis	25	(2.4)
	Other neurological conditions	14	(1.3)
Respiratory tract diseases	Asthma	64	(6.1)
	Other respiratory diseases	45	(4.3)
Other medical conditions	Mass/swelling and other skin conditions	18	(1.7)
	Malignant neoplasms	17	(1.6)
	Other miscellaneous medical conditions	49	(4.7)

**Injuries among patients admitted to public hospitals in Jimma City:** out of the children who had been admitted due to injuries, 625 (62.9%) had unspecified types of injuries. Three hundred and fifty-four (35.6%) were admitted due to unintentional injuries, while only 15(1.5%) were admitted due to intentional injuries. Foreign bodies, poisoning, and burn-related injuries accounted for 116 (11.7%), 41 (4.2%) and 39 (3.9%) of the injury-related admissions, respectively (Table 5).

**Table 5:** injuries among patients admitted to public hospitals in Jimma City, Southwest Ethiopia from September 11^th^, 2014, to September 10^th^, 2017, N=994

Injuries	category	No	(%)
Intentional injuries	Violence related injuries	15	(1.5)
Unintentional injuries	Back and pelvic injuries	5	(0.5)
	Bite injury	34	(3.4)
	Blunt injury	15	(1.5)
	Burn injury	39	(3.9)
	Foreign body	116	(11.7)
	Head/face/neck injury	20	(2)
	Limb injury	20	(2)
	Misc injury	3	(0.3)
	Mva	48	(4.8)
	Poisoning	41	(4.1)
	Stab/cut/open wound	13	(1.3)
Unspecified injuries	Non-specific trauma	625	(62.9)

**Top ten causes of admissions and deaths:** the leading one-third cause of admissions was pneumonia 2274 (29.9 %), followed by an acute febrile illness 626 (8.3%) and non-specific injuries 625 (8.3%). Other pediatric diseases included acute malnutrition 535 (7%) and acute gastro enteritis (AGE) 444 (5.8%) (Table 6). Regarding the causes of death, the largest proportion was largely due to pneumonia (n=36, 22.1%), sepsis (n= 25, 15.3%), and severe acute malnutrition (SAM) (n=25, 15.3%). Related to the year of life lost (PYLL), approximately a total of 9633 years were lost due to premature deaths, of which the majority 7663 (79.6%) were attributed to communicable and nutritional diseases (CNDs), while only 1969.9 (20.4%) of lost years were attributed to non-communicable diseases. Injuries resulted in no PPYLL because no premature death was observed due to injuries. Regarding the specific diseases that were responsible for the highest proportion of PYLL; the top five leading causes of deaths alone were accounted for 66% of the total PYLL occurred in all causes of deaths. Pneumonia was responsible for the highest proportion of PYLL 2178 (22.1%), followed by sepsis 25 (15.3%), SAM 25 (15.3%), diarrhea 15 (9.2%), and meningitis 13 (8%). Approximately 62% of the total years of life lost due to premature deaths were attributed to these top five ranked diseases as shown in (Table 6).

**Table 6:** top ten specific leading causes of admissions and death among pediatric patients admitted to public hospitals in Jimma City, Southwest Ethiopia, from September 11^th^, 2014, to September 10^th^, 2017

	Admission	N=7612		Death	N=163	PYLL (Sum= 9633.4)
Ranks	Top 10 causes	No (%)	Ranks	Top 10 causes	No (%)	No (%)
1	Pneumonia	2274 (29.9)	1	Pneumonia	36 (22.1)	2178.4 (22.6)
2	Acute febrile illness	626 (8.2)	2	Septicemia	25 (15.3)	1492.6 (15.5)
3	Non-specific injuries	625 (8.2)	3	Severe acute malnutrition	25 (15.3)	1478.0 (15.3)
4	Diarrhea	545 (7.2)	4	Diarrhea	15 (9.2)	917.7 (9.5)
5	Sam	535 (7.0)	5	Meningitis	13 (8.0)	753.1(7.8)
6	Septicemia	385 (5.1)	6	Congestive heart failure	9 (5.5)	500.4 (5.2)
7	Meningitis	236 (3.1)	7	Measles	8 (4.9)	467.1(4.8)
8	Measles	218 (2.9)	8	Tuberculosis	6 (3.7)	328.6(3.4)
9	Congestive heart failure	177 (2.3)	9	Congenital anomalies	7 (4.3)	307.0(3.2)
10	Internal abscess	173 (2.3)	10	Intestinal obstruction	3 (1.8)	166.8 (1.7)

## Discussion

This study provides insights into the pattern of admissions and years of potential life lost (YPLL) due to premature death among pediatric admissions in Jimma City public hospitals in southwest Ethiopia. The results of this study indicated that pediatric patients admitted to hospitals were mostly under five years of age, and male predominance was observed, in keeping with findings in other studies carried out elsewhere in Africa [5-8,14-16]. However, this finding is inconsistent with another study conducted in Nigeria, which reported no age or gender variations. This might be due to differences in the population composition of these countries. The great predominance of hospitalizations during the first five years of life could be due to the immunological immaturity of these children; their physiological and physical appearance placed them at a higher risk of acquiring a severe case of disease and getting sick in general [4,17]. The observed male preponderance was also in line with literature that refers to the male gender as a risk factor associated with the hospitalization of children [4,16]. The present study also revealed that children were more likely to be admitted during the wet season than during the dry season. This is similar to the results of another study conducted in Gondar [18]. The findings of this study have shown that, communicable and nutritional diseases were the main reasons for pediatric admissions at Jimma City public hospitals, although the trends decreased over the study period. This finding is in line with the results of other studies conducted in Ethiopia [19,20]. The implication is that less attention is paid to these preventable diseases, which will further affect the health of children if not solved.

The five leading causes of admissions in this study were pneumonia (29.9%), AFI (8.2%), injuries (8.2%), diarrhea (7.2%), and SAM (7%). Whereas another study conducted in Tikur Anbesa Specialized Hospital (TASH) identified the four leading causes of admissions as severe pneumonia (27.5%), heart disease (13.4%), sepsis (8.5%), and diarrhea (7.9%) [21]. The slight differences observed might be due to differences in the study settings. As TASH is the only specialized hospital with cardiac centers in the country, cardiac patients' referral to the hospital seems to be increasing. However, the high SAM burden observed in the present study is consistent with that reported by Gondar study [18]. It is important to note that the causes of admission vary from country to country; for example, while pneumonia was the most common cause of admission in the present study, malaria was the most common cause in a study carried out in Nigeria [14,22]. In this study, the mortality rate was 3%, and the present findings were significantly lower than that of several other studies conducted elsewhere in the world [6,8,14,23-25]. But this is relatively consistent with the rate found in the previous study reported by Osarumwense *et al*., which was 3.1% [26] and higher than the other studies conducted by Elmogy *et al*. [7]. This indicates that there is significant variation among mortality rates throughout the world, which could be due to the differences in geographical location, duration of study periods, disease burden, pathologic diversity, type of hospital, and methodology, as some studies were conducted in neonatal children, while some were conducted in specific pediatric units such as pediatric emergency and pediatric surgical units.

The current study showed that the top five causes of death were pneumonia, septicemia, SAM, diarrhea, and meningitis. This finding is consistent with the Ethiopian Demographic Health Survey (EDHS), 2019 report [2] and other similar reports throughout Africa [6,14,18,22,25]. However, this finding differs from findings from the developed world, which reported that the leading causes of death in children were non-communicable diseases such as neoplasms, injuries (road injuries, drowning, suicide, homicide, and other injuries), congenital anomalies, and heart diseases [27-29]. The difference from the current study might be due to a change in disease patterns from communicable to non-communicable diseases in the developed countries and also in these countries, there are better controls over the infectious agent by utilizing better disease prevention and control mechanisms. Concerning potential years of life lost due to premature death (PYLL), in the present study, 9633 years of life were lost due to the causes of death identified in this study. Years of life lost from communicable diseases predominately occurred due to infectious and parasitic diseases (44.7%) and respiratory diseases (22.6%). Among non-communicable diseases, cardiovascular disease accounted for PYLL of 6.4%). For specific diseases in this study, the greater percentage of PYLL was due to pneumonia (22.6%), followed by sepsis (15.5%) and SAM (15.3%). This is consistent with the Global burden of disease tudy 2010 (GBD 2010) and the GBD 2017 report, which showed that lower respiratory infections and diarrheal diseases were the highest-ranking causes of PYLL due to premature death in Ethiopia [3,12,30].

In contrast to this finding, studies conducted in the USA and New England revealed that central nervous system's tumors and unintentional injury were the major causes of PYLL (31%) [31,32], suicides, traffic accidents, cardiovascular diseases in Poland [33,34], listeriosis in Germany [35], cardiovascular diseases and cancers dominated the burden of premature mortality in Serbia [36], injuries both in the developing and developed world [37], cancer in Japan [38], accidents and injury in Australia [39], and cancer in New Jersey [40]. This indicates that the leading cause of PYLL in the developed world is a non-communicable disease. The observed differences could be explained by Geographical locations, time, and demographics (some studies were carried out among post pediatric age group) and methodology, as some studies have been conducted on a specific disease such as cancers. The reason for this difference could also be due to a change in disease patterns from communicable to non-communicable diseases in the developed world. A merit of this study is that it studied all children admitted to the pediatric wards of the two hospitals within three years without sampling, hence eliminating any possible sampling error. However, the findings of this study should be interpreted in light of its limitations. Although a hospital-based study provides a means to examine admissions and mortality in the hospital setting and reflects the probable state of diseases, it still lacks representation of its outcome to the general population. Furthermore, a lack of information on comorbidities may affect the assessment of the outcomes of various diseases.

## Conclusion

This study indicates that the leading causes of hospital admissions, deaths, and PYLLs were communicable and nutritional diseases such as pneumonia, sepsis, severe acute malnutrition, diarrhea, and meningitis, which are preventable and curable with available medical technology. There is a possibility to reduce childhood morbidity and mortality by improving timely case detection and management. Therefore, it is recommended that early detection of these diseases and timely initiation of an appropriate intervention could reduce hospital burden and the years of life lost from them. Moreover, further community-based interventional studies should be conducted on a larger scale to broaden the understanding gained from this initial study.

### What is known about this topic

- The trends in pediatric admissions and mortality are increasing over years;
- The burden of preventable causes of deaths and admissions in children is increasing, but has not gained much attention in Africa.

### What this study adds

- Premature mortality among pediatric patients is mostly due to a communicable and nutritional diseases;
- Approximately 62% of the total potential years of life lost due to premature deaths were attributed to pneumonia, sepsis, severe acute malnutrition, diarrhea and meningitis;
- Pneumonia accounted for the highest proportion of potential years of life lost in pediatric patients.
